# Colorectal cancer screening behaviors of general surgeons and first-degree family members: a survey-based study

**DOI:** 10.1186/s12876-019-1106-x

**Published:** 2019-11-12

**Authors:** Suleyman Utku Celik, Hasan Gorkem Cay, Ersin Bayrakdar, Aysima Ince, Esra Nur Ince, Yasemin Celik, Yunus Emre Yucel, Mehmet Ali Koc, Siyar Ersoz, Cihangir Akyol

**Affiliations:** 1 Department of General Surgery, Gulhane Training and Research Hospital, Ankara, Turkey; 2 Department of General Surgery, Ankara University School of Medicine, Ankara, Turkey

**Keywords:** Attitude, Behavior, Colorectal cancer, General surgeons, Screening

## Abstract

**Background:**

Colorectal cancer (CRC) screening rates are low in the general population and among health care providers. The aim of this study was to evaluate the CRC screening practices of general surgeons who provide specialized diagnostic testing and CRC treatment and to examine the CRC screening behaviors of their first-degree family members.

**Methods:**

A cross-sectional survey was conducted among general surgeons who attended the 21st National Surgical Congress in Turkey held from April 11th to 15th, 2018. The survey included items on demographics, screening-related attitude, CRC screening options, barriers to CRC screening, and surgeons’ annual volumes of CRC cases.

**Results:**

A total of 530 respondents completed the survey. Almost one-third of the responding surgeons (29.4%, *n* = 156) were aged over 50 years, among whom approximately half (47.1%, *n* = 74) reported having undergone CRC screening and preferring a colonoscopy as the screening modality (78.4%). Among general surgeons aged 50 years and older, high-volume surgeons (≥25 CRC cases per year) were more likely to undergo screening compared with low-volume surgeons (< 25 CRC cases per year). The respondents aged below 50 years reported that 56.1% (*n* = 210) of their first-degree relatives were up-to-date with CRC screening, mostly with colonoscopy. Compared to low-volume surgeons aged below 50 years, high-volume surgeons’ first-degree relatives were more likely to be up-to-date with CRC screening.

**Conclusion:**

The survey results demonstrated that routine screening for CRC among surgeons and/or their first-degree relatives is currently not performed at the desired level. However, high-volume surgeons are more likely to participate in routine screening.

## Background

Colorectal cancer (CRC) screening is performed to diagnose early-stage CRC and precancerous lesions in asymptomatic individuals. Currently, the US Multi-Society Task Force of Colorectal Cancer and US Preventive Services Task Force (USPSTF) recommend CRC screening to be initiated at 50 years of age and to continue the screenings until 75 years of age for individuals at average risk of the disease [1, 2]. Furthermore, the USPSTF recommends screening for eligible individuals using stool-based screening tests such as fecal immunochemical tests (annually), guaiac-based fecal occult blood tests (FOBTs) (annually), and multitarget stool DNA tests (every 1 or 3 years) or tests that directly and effectively visualize the colon, including colonoscopy (every 10 years), flexible sigmoidoscopy (every 5 years), and computed tomography colonography (every 5 years) [1, 2]. In 2014, the National Colorectal Cancer Roundtable, co-founded by the American Cancer Society (ACS) and the Centers for Disease Control and Prevention, announced an initiative named “80% by 2018.” This initiative set the major public health goal of screening 80% of eligible adults aged 50 years and older by 2018 to reduce the incidence and mortality rates from CRC by over 20 and 33%, respectively, by 2030 [3, 4]. Despite the relatively high availability of tests, widespread campaigns, and educational efforts, a significant proportion of adults aged 50–70 years remain unscreened; in 2016, only 67.3% of eligible adults were screened for CRC in the United States [2, 5, 6]. Overall, CRC screening rates are unacceptably low, especially in undeveloped and developing countries as well as in most European countries [6–8].

General surgeons involved in screening, management, and follow-up of CRC treatment as well as in policy initiatives, national cancer prevention campaigns, and community awareness activities might be eligible for CRC screening and/or have eligible family members aged 50 years and older. However, CRC screening rates are low in the general population and among health care providers [9, 10]. The aim of this study was to evaluate the CRC screening practices of general surgeons who provide specialized diagnostic testing and CRC treatment and to examine the CRC screening behaviors of their first-degree family members.

## Methods

### Individuals

A cross-sectional survey was administered to each registered Turkish general surgeon attending the 21st National Surgical Congress, the largest and most representative national meeting of general surgeons, at Antalya, Turkey (April 11–15, 2018). Surgeons who did not complete the questionnaire were excluded from the study.

### Questionnaire

Survey questions related to CRC screening practices were designed in accordance with the USPSTF recommendations [2]. To assess CRC screening behaviors, the CRC screening status of the surgeons and their relatives was evaluated.

The questionnaire included closed-ended questions. The first part of the questionnaire consisted of the demographic details of the surgeons, which included age, gender, workplace, academic title, and annual volumes of CRC cases. Here, the respondents were divided into two groups according to their age: (1) aged below 50 years and (2) aged 50 years and older. In the second part, for the surgeons assigned to Group 1, the questionnaire included questions on their attitude related to screening for their first-degree relatives (mother or father) and their screening options; for the surgeons assigned to Group 2, the questionnaire comprised questions regarding CRC screening for themselves, barriers to screening, and family history.

Content validity of the questionnaire was assessed by five experts in the field of colorectal surgery to ensure its suitability for the study, to edit questions for clarity and to remove redundant questions. Then, the questionnaire was piloted with a sample of 10 general surgeons from the researchers’ institute to ensure the clarity of its items.

### Surgeon volume

A surgeon performing 25 or more CRC operations annually was classified as a high-volume surgeon and a surgeon who performed on average fewer than 25 cases annually was classified as a low-volume surgeon. This categorization was made in accordance with recent studies [11–13].

### Data collection

Data were collected with self-administered, anonymous, multiple-choice questionnaires (Additional file 1). The information gathering instrument created by the research team was a web-based survey with a total of eleven questions.

The final instrument was delivered to the participants via a web-based survey platform after obtaining permission from the chair of the congress committee. In addition to recurring emails, reminders were sent to participants at regular intervals if they did not complete the survey within the allocated time. A maximum of two reminders were sent to nonresponders, 1 day and 3 days after the initial invitation. After 4 days, the survey was set offline and the data were locked.

### Statistical analysis

Descriptive analysis was used to assess the demographic details of the study participants. Data were expressed as frequencies (%) for categorical variables. The differences in terms of proportion between the groups were compared using the chi-square or Fisher’s exact test, where appropriate. A *p*-value of < 0.05 was considered statistically significant. Missing data were handled by listwise deletion for each analysis. All statistical data were analyzed using the Statistical Package for the Social Sciences program (version 16.0 for Windows; Chicago, IL).

### Ethical approval

This study was approved by the Ankara University School of Medicine Undergraduate Student Researches Ethics Committee (approval number: 72189195–050.03.04-E.6039) and was carried out in accordance with the 1964 Helsinki Declaration and its later amendments. In addition, written informed consent for publication of individuals’ clinical details was obtained.

## Results

The survey was conducted among 553 general surgeons. Twenty-three surgeons who did not complete the questionnaire were excluded from the study. Therefore, a total of 530 questionnaires were completed, with an overall response rate of 95.8%. The respondents were divided into two groups according to their age: (1) aged below 50 years (Group 1, *n* = 374) and (2) aged 50 years and older (Group 2, *n* = 156). Table 1 presents the CRC screening data for the first-degree relatives of the surgeons aged below 50 years, whereas Table 2 presents the self-screening data of the surgeons aged 50 years and older.

**Table 1:** Screening characteristics of first-degree relatives of surgeons younger than 50 years of age

Surgeon characteristics	CRC screening for surgeons’ first-degree relatives		*P*-value
	No (*n* = 164)	Yes (*n* = 210)	
Sex, n			
Male	141 (42.2%)	193 (57.8%)	0.066
Female	23 (57.5%)	17 (42.5%)	
Workplace, n			
State hospital	48 (44.4%)	60 (55.6%)	0.853
Private hospital	19 (44.2%)	24 (55.8%)	
Training and research hospital	58 (46.0%)	68 (54.0%)	
University hospital	39 (40.2%)	58 (59.8%)	
Academic title, n			
Specialist	119 (46.9%)	135 (53.1%)	0.179
Associate professor	36 (36.0%)	64 (64.0%)	
Professor	9 (45.0%)	11 (55.0%)	
Volume of CRC cases, n			
Low volume (< 25 cases)	117 (48.1%)	126 (51.9%)	0.023
High volume (≥25 cases)	47 (35.9%)	84 (64.1%)	

*CRC* Colorectal cancer

**Table 2:** Screening characteristics of surgeons aged 50 years and older

Surgeon characteristics	CRC screening status of the surgeons		*P*-value
	No (*n* = 82)	Yes (*n* = 74)	
Sex, n			
Male	79 (52.7%)	71 (47.3%)	1.000
Female	3 (50.0%)	3 (50.0%)	
Workplace, n			
State hospital	20 (55.6%)	16 (44.4%)	0.807
Private hospital	15 (50.0%)	15 (50.0%)	
Training and research hospital	24 (57.1%)	18 (42.9%)	
University hospital	23 (47.9%)	25 (52.1%)	
Academic title, n			
Specialist	45 (60.0%)	30 (40.0%)	0.067
Associate professor	11 (61.1%)	7 (38.9%)	
Professor	26 (41.3%)	37 (58.7%)	
Volume of CRC cases, n			
Low volume (< 25 cases)	60 (59.4%)	41 (40.6%)	0.020
High volume (≥25 cases)	22 (40.0%)	33 (60.0%)	
Family history, n			
No	76 (57.6%)	56 (42.4%)	0.003
Yes	6 (25.0%)	18 (75.0%)	

*CRC* Colorectal cancer

The majority of the surgeons were males (*n* = 484, 91.3%). Among the general surgeons aged 50 years and older, 47.1% (*n* = 74) had previously undergone screening for CRC in a timely manner. The CRC screening methods preferred by surgeons included colonoscopy and FOBT (78.4 and 17.6%, respectively). Surgeons who had been screened for CRC exhibited no differences in terms of sex (*p* = 1.000), workplace (*p* = 0.807), or academic title (*p* = 0.067) compared with those who had not been screened. However, surgeons handling over 25 CRC cases annually (high-volume surgeons) were more likely to undergo screening for themselves compared with those handling less than 25 CRC cases annually (low-volume surgeons) (60.0% vs. 40.6%, *p* = 0.020). Expectedly, surgeons with a family history of CRC were more likely to adhere to screening recommendations than those without a family history (*p* = 0.003). The main barriers to undergoing CRC screening included procrastination owing to work intensity (63.4%), the belief that CRC screening is not necessary (21.9%), and anxiety or fear related to CRC screening (14.7%).

Among the 374 surgeons aged below 50 years, slightly more than half (56.1%) reported that at least one of their first-degree family members were up-to-date with the CRC screening recommendations. Colonoscopy was the most preferred (78.1%) screening tool. Notably, there were no significant differences in terms of sex (*p* = 0.066), workplace (*p* = 0.853), and academic title (*p* = 0.179) between surgeons whose first-degree relatives had undergone CRC screening and those whose relatives had not undergone the screening. Similarly, compared to low-volume surgeons, high-volume surgeons’ first-degree relatives were more likely to be up-to-date with CRC screening of any type (51.9% vs. 64.1%, *p* = 0.023).

## Discussion

Although CRC screening rates are modestly improving, we are still a long way from the National Colorectal Cancer Roundtable’s goal of 80% of adults aged 50 and older being regularly screened for CRC by 2018 [3, 4]. Unfortunately, the data from this study revealed that more than half of surveyed surgeons aged 50 years and older had not previously been screened for CRC in a timely manner even though national CRC screening policies and guidelines may be in place. Despite differences in the perceived barriers between surgeons and the general population, our results are important in the context of understanding the barriers to CRC screening. Understanding the reason why health care providers or surgeons, who routinely provide screening options to their patients and have a broad knowledge of CRC and its risks, do not follow the recommendations for routine screening for themselves is as valuable as other policies and campaigns developed for CRC screening. The primary purpose of this study was to evaluate the CRC screening practices of general surgeons and their first-degree family members. We also assessed the main barriers to routine CRC screening for themselves.

Globally, CRC is the third most commonly diagnosed cancer among males and females [14, 15]. The incidence and mortality rates of CRC have been decreasing over the past several decades due to changes in related risk factors, an increase in routine screening facilitating precursor lesion removal, and advances in surgical and medical management. Because most CRC develops slowly from removable precancerous lesions known as adenomas, which can take up to 10 years or more to become malignant, detection of early-stage CRC has a good prognosis [16, 17]. Therefore, CRC is a suitable candidate for screening to markedly reduce the incidence and mortality of the disease [1, 5, 17, 18]. Furthermore, CRC screening by any currently available modality appears to be more cost-effective than not performing screening and the cost burden of CRC treatment [1, 18].

Regular CRC screening is vital for the early detection of precursor lesions in asymptomatic individuals aged 50–75 years [1, 2]. Numerous CRC deaths could also be prevented through increased population-based screening programs [1, 5, 14, 17, 18]. Although multiple screening options are available, there is no evidence that one screening test is superior to another; in fact, all tests have a comparable ability to reduce CRC mortality rates via early detection, provided average-risk adults adhere to the steps mentioned in the screening guidelines at the appropriate time intervals [17, 19, 20]. However, despite the availability of these tests, widespread recommendations for screening, and the presence of screening programs, nearly one-quarter of eligible adults have never been screened for CRC; in 2016, only 67.3% of age-appropriate individuals were updated about screening in the United States [2, 5, 6]. This statistic is not better in other countries; in fact, it is worst in developing countries. The screening rates of European countries range from 20 to 68%, which is far from the targeted goals and desirable standards [7, 21].

Provider adherence to evidence-based screening guidelines is crucial for CRC prevention and control, regardless of health policy initiatives and campaigns [22]. Provider recommendations and guidance can also improve compliance with CRC screening [1, 6]. In Turkey, general surgeons and gastroenterologists are the major providers of gastrointestinal (GI) endoscopy and other specialized screening options. Although many studies emphasized that CRC screening is recommended to average-risk adults by most health care providers (65–95%), personal routine CRC screening rates are low in those deemed eligible for screening [9, 10, 23, 24]. In a study by Terhaar Sive Droste et al., this difference was highlighted to be more pronounced when compared between GI specialists (gastroenterologists and gastrointestinal surgeons) and general practitioners (GPs). It was found that screening for CRC was strongly supported by Dutch GI specialists and less by GPs. Moreover, a significant difference was found in the preferred personal screening method among them. While screening with colonoscopy was favored by 97% of GI specialists, only 27% of GPs preferred this method [25].

To the best of our knowledge, this study is the first in the English literature to examine general surgeons’ personal routine CRC screening practices. The study focused on the personal or first-degree relatives’ CRC screening practices of 530 general surgeons who routinely provide screening options to their patients at average risk for CRC in Turkey. In the present study, we found that less than half (47.1%) of the surgeons aged 50 years and older had previously been screened for CRC in a timely manner, and only half (56.1%) of the surgeons under 50 years of age reported that their first-degree family members were up-to-date with CRC screening. Similarly, in a study conducted by Rim et al., which included 109 health care professionals such as family physicians, internists, physician assistants, and nurse practitioners aged over 50 years, just over half (60%) had been screened for CRC and slightly less than half (48%) were not up-to-date with CRC screening recommendations [9]. Our data also showed that surgeons with a positive family history of CRC were more motivated to screen for CRC. However, the workplace or academic title of the surgeons had no impact on the routine screening practice of the surgeons or their family members.

It is well known that surgical specialization and high-volume practice are both associated with better outcomes for CRC surgery. However, there is no definite cut-off value of surgeon volume at which to decide whether a surgeon is a high- or low-volume surgeon. In addition, this value changes according to country, city, and hospital [11–13]. In our study, this categorization was made in accordance with recent studies, and the surgeons were grouped into two categories (low volume: < 25 cases; high volume: ≥25 cases). Our data showed that compared with low-volume surgeons aged 50 years and older, high-volume surgeons were more likely to adhere to screening recommendations and similarly, compared with low-volume surgeons under 50 years of age, the first-degree relatives of high-volume surgeons were significantly more likely to be up-to-date with CRC screening. These results suggest that general surgeons familiar with CRC cases or specifically interested in colorectal surgery may recognize the benefits of early screening for CRC in particular compared with those who are not.

For the general population, poor awareness of CRC and screening programs, characteristics of screening tests such as patient preparation or complications for colonoscopy and the potential to overlook cancer or a false positive result for FOBT, lack of time, and financial issues are important factors that affect the attendance rate of CRC screening programs [18, 23, 26]. However, the perceived barriers for health care providers are significantly different from the general population. In this study, the main barrier to screening was found to be procrastination owing to work intensity (63.4%); the other barriers were the belief that CRC screening is not necessary, worry about the results, anxiety, and fear about complications. In addition, while female physicians are far more likely to recommend screening compared to their male counterparts [24, 27], female surgeons at average risk for CRC in our study were not more likely to be up-to-date with CRC screening guidelines than males. Based on our survey results, it is difficult to explain the lower screening rates of general surgeons and their first-degree family members. Although not exactly the same, the general population and surgeons probably have similar concerns with regard to CRC screening. However, health care providers who routinely provide screening options to their patients at average risk for CRC differ from the general population due to the nature of their work. Therefore, expecting the general population to adhere to CRC screening recommendations that health care providers themselves do not follow and trying to increase the CRC screening rates only through campaigns and national screening policies is unrealistic. We think that provider adherence to evidence-based guidelines for themselves and for their relatives as well as innovative strategies for improving CRC screening are crucial for achieving screening goals.

Despite the strength of a large sample size of general surgeons with a high response rate, several limitations need to be considered. First, the current study relied on self-reported data. Thus, reporting and recall bias is possible. Selection bias represents another potential limitation because the enrolled surgeons were selected from those surgeons who attended a national congress in Turkey. One other limitation is that the questionnaire contained ‘closed-ended’ questions. Last, the chosen threshold for high-volume surgeons was determined according to recent studies rather than nationwide surgical data.

## Conclusions

Even general surgeons involved in the treatment of patients with CRC neglect to participate in CRC screenings, and the screening rate of their first-degree relatives is similar to that of the general population, i.e., not at the desired level. However, high-volume surgeons are more likely to undergo screening than low-volume surgeons. Because an increase in CRC screening rates largely depends on the efforts of health care providers, such as general surgeons, provider-related barriers to CRC screening need to be tackled to achieve acceptable screening rates.

## Additional file

**Additional file 1.** Survey questions related to colorectal cancer screening behaviors of general surgeons and first-degree family members.

## Data Availability

The datasets generated and/or analyzed during the current study are not publicly available but are available from the corresponding author on reasonable request.

## Abbreviations

ACS: American Cancer Society
CRC: Colorectal cancer
FOBT: Fecal occult blood test
GI: Gastrointestinal
GP: General practitioner
USPSTF: US Multi-Society Task Force of Colorectal Cancer and US Preventive Services Task Force
