# Supplementary material for: Colorectal cancer screening behaviors of general surgeons and first-degree family members: a survey-based study
Source: BMC Gastroenterol. 2019 Nov 12;19:183. doi: 10.1186/s12876-019-1106-x (PMC6852782; doi:10.1186/s12876-019-1106-x)
Supplement: Supplementary file 1 — Additional file 1. Survey questions related to colorectal cancer screening behaviors of general surgeons and first-degree family members. [file 12876_2019_1106_MOESM1_ESM.docx]

**Appendix 1.** Survey questions related to colorectal cancer screening behaviors of general surgeons and first-degree family members

1. **Age:** (a) < 50 (b) ≥ 50
2. **Gender:** (a) Female (b) Male
3. **Workplace:** (a) State hospital

(b) Private hospital

(c) Training and research hospital

(d) University hospital

1. **Academic title:** (a) Specialist (b) Associate professor (c) Professor
2. **Annual volume of CRC cases:** (a) < 25 cases (b) ≥ 25 cases

*Questions for individuals aged below 50 years*

1. **Is at least one of your first-degree family members up-to-date with the CRC screening recommendations?**

(a) No (b) Yes

1. **If yes, which of the following screening tests were preferred to screen your relatives?**

(a) Fecal occult blood test (within this year)

(b) Sigmoidoscopy (within 5 years)

(c) Colonoscopy (within 10 years)

(d) Computed tomography colonography (within 5 years)

*Questions for individuals aged 50 years and older*

1. **Do you have any family history of CRC?**

(a) No (b) Yes

1. **Are you up-to-date with the CRC screening recommendations?**

(a) No (b) Yes

1. **If yes, which of the following screening tests were preferred?**

(a) Fecal occult blood test (within this year)

(b) Sigmoidoscopy (within 5 years)

(c) Colonoscopy (within 10 years)

(d) Computed tomography colonography (within 5 years)

1. **If no, what is the main barrier to undergoing CRC screening?**

(a) Procrastination owing to work intensity

(b) The belief that CRC screening is not necessary

(c) Worry about results

(d) Anxiety

(e) Fear about complication

(f) Other …………

| CRC, colorectal cancer. |
| --- |
